# LncRNA SNHG1 was down-regulated after menopause and participates in postmenopausal osteoporosis

**DOI:** 10.1042/BSR20190445

**Published:** 2019-11-12

**Authors:** Shuaihao Huang, Xiaowen Zhu, Dan Xiao, Jianxiong Zhuang, Guoyan Liang, Changxiang Liang, Xiaoqing Zheng, Yuhong Ke, Yunbing Chang

**Affiliations:** Department of Orthopedics, Guangdong General Hospital, Guangdong Academy of Medical Sciences, Guangzhou City 510080, Guangdong Province, P.R. China

**Keywords:** diagnosis, lncRNA SNHG1, postmenopausal osteoporosis

## Abstract

The functions of long (>200 nt) non-coding RNA (lncRNA) small nucleolar RNA host gene 1 (SNHG1) have only been investigated in cancer biology. We found that plasma LncRNA SNHG1 was down-regulated in postmenopausal than in premenopausal females. Among postmenopausal females, the ones with postmenopausal osteoporosis showed much lower expression levels of plasma lncRNA SNHG1. A 6-year follow-up study on postmenopausal females revealed that plasma lncRNA SNHG1 decreased in females with postmenopausal osteoporosis but not in healthy postmenopausal females. Levels of plasma lncRNA SNHG1 at 12 months before diagnosis is sufficient to distinguish postmenopausal osteoporosis patients from healthy controls. After treatment, plasma lncRNA SNHG1 were significantly up-regulated. Therefore, lncRNA SNHG1 was down-regulated after menopause and plasma level of lncRNA SNHG1 may serve as a biomarker for the diagnosis and treatment of postmenopausal osteoporosis.

## Introduction

Menopause causes a series of changes in the females, such as difficulty in sleeping, weight gain, mood disturbances, and reduced productions of certain hormones, such as estrogen synthesized in ovary [1]. Reduction in estrogen production leads to the loss of bone minerals, resulting in osteoporosis [2]. The development of osteoporosis increases the risk of bone fractures [3]. Postmenopausal osteoporosis affects more than half of the postmenopausal females [4,5]. Decreased estrogen production causes imbalance between bone formation by osteoblasts and bone resorption by osteoclasts, which is the major contributor of postmenopausal osteoporosis [6]. However, the molecular pathways involved in postmenopausal osteoporosis still have not been fully elucidated.

Long (>200 nt) non-coding RNAs (lncRNAs), are critical determinants in diverse physiological and pathological processes [7–9]. LncRNAs achieve their functions mainly by regulating downstream genes at translational and post-transcriptional levels or through epigenetic pathways [10]. Therefore, altered lncRNA levels may result in abnormal expression of genes involved in human diseases [9], and regulation of lncRNA expression have been proved as promising therapeutic target for human diseases [11]. However, function of most lncRNAs remains unknown. LncRNA small nucleolar RNA host gene 1 (SNHG1) is a well-studied oncogenic lncRNA [12,13], while its role in other diseases is unknown. Our RNA-seq data revealed that SNHG1 was down-regulated in postmenopausal osteoporosis (data not shown), indicating its involvement in this disease. Our study was performed to explore the clinical value of SNHG1 for postmenopausal osteoporosis.

## Materials and methods

### Patients

A total of 122 healthy premenopausal females and 278 postmenopausal females were enrolled in Guangdong General Hospital from March 2010 to March 2012. None of the 122 premenopausal females were diagnosed with osteoporosis. Among the 278 postmenopausal females, 76 cases were diagnosed with postmenopausal osteoporosis, and the rest 202 cases were healthy postmenopausal females. The diagnosis of osteoporosis was perfromed according to the following criteia: more than 2.5 SD decrease in BMD compared with healthy poplation. Patients with other clinical complications were excluded from the present study. None of the participants received any treatments within 3 months before admission. Age of the 122 healthy premenopausal females ranged from 32 to 48 years, with a mean age of 41.1 ± 4.9 years, T score ranged from −0.8 to 3.1, with a mean of 1.2 ± 0.4. Age of 202 healthy postmenopausal females ranged from 51 to 72 years, with a mean age of 62.3 ± 4.5 years, T-score ranged from −0.9 to 3.0, with a mean of 1.3 ± 0.5. Age of the 76 postmenopausal osteoporosis patients ranged from 52 to 70 years, with a mean age of 63.3 ± 4.7 years, T-score of the patients ranged from −2.6 to −4.8, with a mean of −3.4 ± 0.6, disease duration ranged from 2.5 to 10.8 years, with a mean of 7.1 ± 2.8 years. The formula to calculate T-score was: T-score = (bone mineral density − reference bone mineral density)/reference standard deviation. The T-score was significantly lower in patient group than in other two groups. No significant differences in age was found between healthy postmenopausal females and postmenopausal osteoporosis patients, while healthy premenopausal females were younger that other two groups of participants. The present study had been approved by the Ethics Committee of Guangdong General Hospital before admission of participants. All participants signed informed consent.

### Treatment, follow-up, and plasma samples

Blood was extracted from all participants under fasting conditions before any treatments. The 76 postmenopausal osteoporosis patients were treated with bisphosphonates (such as alendronate and risedronate), or hormones (such as estrogen), or other medications (such as denosumab) or the combination of them according to their individual conditions. Blood was also extracted from the 76 postmenopausal osteoporosis patients at 3 months after the beginning of treatment. The 202 healthy postmenopausal females were followed up (outpatient visit, once per half year) for 6 years after admission to record the occurrence of osteoporosis. Blood was extracted during every outpatient visit. Blood was transferred to EDTA-treated tubes and centrifuged at 1400×***g*** for 10 min to prepare plasma.

### RNA preparation and RT-qPCR

RNAzol reagent (Sigma–Aldrich, St. Louis, MO, U.S.A.) was mixed with plasma to extract total RNAs. Total RNAs were reverse-transcribed to synthesize cDNA using SSRT II system (Thermo Fisher Scientific) by incubating at 25°C for 5 min, 55°C for 10 min, and 75°C for 5 min. To detect the expression of SNHG1, Applied Biosystems™ PowerUp™ SYBR™ Green Master Mix was used to prepare PCR systems, and PCRs were perfromed with 18S RNA as endogenous control. Primer sequences were: 5′-AGGCTGAAGTTACAGGT-3′ (forward) and 5′-TTGGCTCCCAGTGTCTT-3′ (reverse) for SNHG1; 5′-TACCACATCCAAGGAAGCA-3′ (forward) and 5′-TTTTCGTCACTACCTCCCCG-3′ (reverse) for 18S rRNA. Expression of SNHG1 of normalized to 18S RNA according to 2^−ΔΔ*C*_T_^ method. PCR products were sequenced to make use corrected products were obtained. There replicates were performed for each reaction.

### Statistical analysis

Data of three biological replicates were used to calculate mean ± standard deviation values. GraphPad Prism 6 software was used to process data and perform all statistical analyses. Comparisons among different groups of participants were performed by ANOVA (one-way) and Tukey’s test. Comparisons between pre- and post-treatment levels of SNHG1 were performed by paired *t* test. ROC curve analysis was performed to evaluate the diagnostic values of plasma SNHG1 for postmenopausal osteoporosis with postmenopausal osteoporosis patients as true positive cases and healthy females as true negative cases. Differences with *P*<0.05 were statistically significant.

## Results

### Plasma levels of SNHG1 were affected by both menopause and osteoporosis

**Figure 1:**
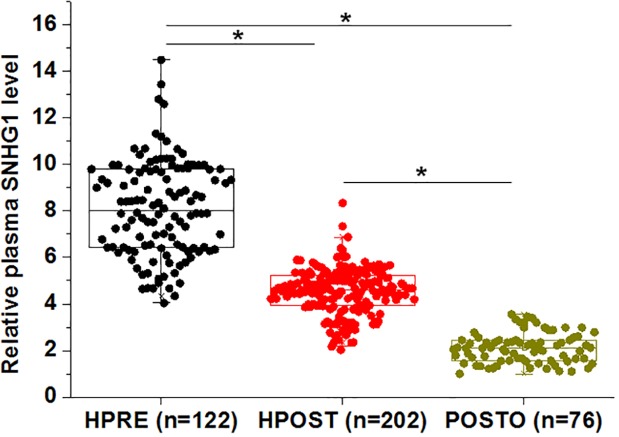
Plasma levels of SNHG1 was affected by both menopause and osteoporosis Compared with 122 healthy premenopausal females (HPRE group), plasma levels of SNHG1 were reduced in 76 postmenopausal osteoporosis patients (POSTO group) and 202 healthy postmenopausal females (HPOST group). Compared with HPOST group, plasma levels of SNHG1 were further reduced in POSTO group (*, *P*<0.05).

RT-qPCR was performed to investigate the differential expression of SNHG1 in the 122 healthy premenopausal females (HPRE group), 76 postmenopausal osteoporosis patients (POSTO group), and 202 healthy postmenopausal females (HPOST group). Compared with patients in HPRE group, plasma levels of SNHG1 were significantly lower in POSTO and HPOST groups. In addition, compared with HPOST group, plasma levels of SNHG1 were further reduced in POSTO group (Figure 1, *P*<0.05).

### Plasma levels of SNHG1 decreased in females who developed during postmenopausal osteoporosis

**Figure 2:**
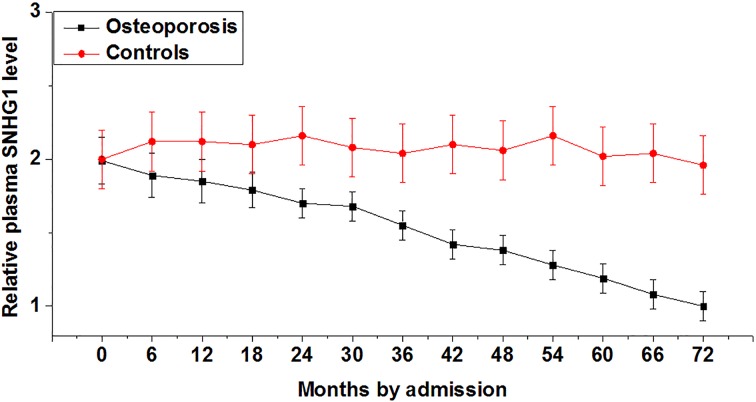
Plasma levels of SNHG1 decreased in females who developed during postmenopausal osteoporosis A 6-year follow-up study revealed that plasma levels of lncRNA SNHG1 decreased obviously in females with postmenopausal osteoporosis but not in healthy postmenopausal females.

During the 6-year follow-up, 67 out of the 202 healthy postmenopausal females developed osteoporosis. As shown in Figure 2, plasma levels of SNHG1 decreased obviously in females with postmenopausal osteoporosis but not in healthy postmenopausal females.

### Plasma levels of SNHG1 at 12 months before diagnosis distinguished postmenopausal osteoporosis patients from healthy controls

**Figure 3:**
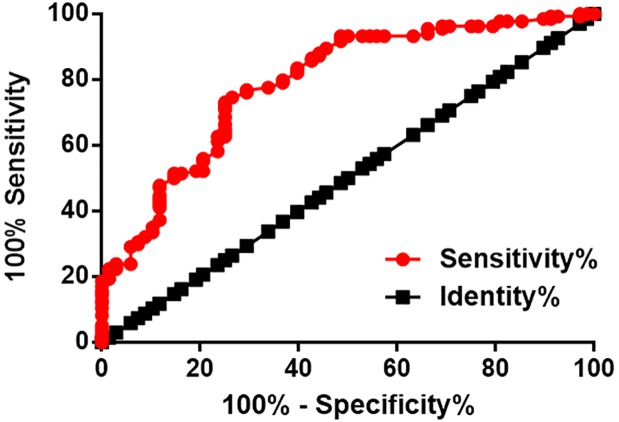
Plasma levels of SNHG1 at 12 months before diagnosis distinguished postmenopausal osteoporosis patients from healthy controls ROC curve analysis showed that plasma levels of SNHG1 at 12 months before diagnosis is sufficient to distinguish postmenopausal osteoporosis patients from healthy controls.

ROC curve analysis was performed. As shown in Figure 3, area under the curve (AUC) of the use of plasma levels of SNHG1 at 12 months before diagnosis was 0.79, with standard error of 0.034 and 95% confidence interval of 0.72–0.86. In addition, plasma levels of SNHG1 early than this time point failed to provide an AUC larger than 0.65.

### Plasma levels of SNHG1 were significantly increased after treatment

**Figure 4:**
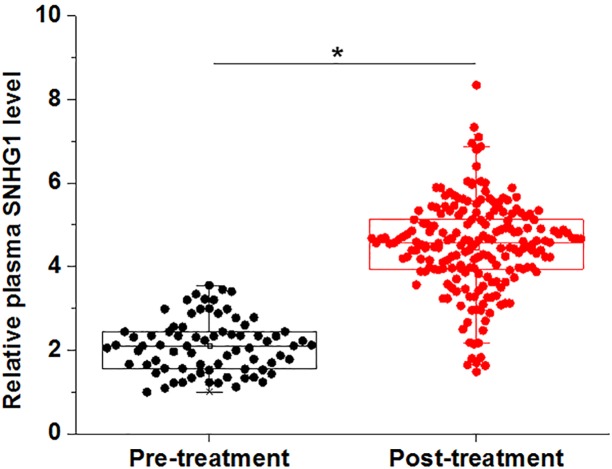
Plasma levels of SNHG1 were significantly increased after treatment Plasma SNHG1 were significantly up-regulated in the 76 postmenopausal osteoporosis patients at 3 months after the beginning of treatment (post-treatment) compared with pre-treatment levels (*, *P*<0.05).

Compared with the pre-treatment levels of plasma SNHG1, plasma levels of SNHG1 were significantly increased in the 76 postmenopausal osteoporosis patients at 3 months (post-treatment) after the beginning of treatment (Figure 4, *P*<0.05).

## Discussion

SNHG1 is a well-studied oncogenic lncRNA in different types of cancer [12,13]. We, in the present study, first showed that SNHG1 was down-regulated with menopause and the development of osteoporosis, and plasma SNHG1 showed predictive values for postmenopausal osteoporosis.

Microarray studies have revealed that the development of postmenopausal osteoporosis is accompanied with globally altered expression of lncRNAs [14], indicating the involvement of lncRNAs in this diseases. Although natural menopause usually causes no obvious physical changes, transcriptome analysis has shown that natural menopause can lead to the dysregulated expression of a large set of genes [15]. Our RNA-seq data revealed that SNHG1 was down-regulated in postmenopausal osteoporosis (data not shown). Therefore, SNHG1 may be involved in the development of osteoporosis. In the present study we observed that plasma SNHG1 was down-regulated with menopause and the development of osteoporosis. Therefore, menopause may mediate the down-regulation of SNHG1, which contributes to the development of osteoporosis. However, molecular mechanism of the involvement of SNHG1 in this disease is still unknown. Natural menopause causes altered expression of progesterone, testosterone and estrogen, while these hormones, such as estrogen can regulate the expression of certain lncRNAs [16,17]. In addition, the development of osteoporosis after menopause is also affected by dysregulated expression of hormones [18]. Therefore, the down-regulated SNHG1 after menopause and during the development of osteoporosis may be also caused by the changed levels of hormones. Our future studies will explore the interactions between SNHG1 and hormones. As an oncogenic lncRNA, SNHG1 is overexpressed in different types of cancer [12,13]. Therefore, the expression pattern of SNHG1 can be different in different types of diseases.

The incidence of osteoporosis is unacceptably high among postmenopausal females. It has been reported that the long-term consumption of calcium and vitamin K_2_ can result in reduced incidence rate of postmenopausal osteoporosis [19,20]. However, the long-term use of supplements is not practical in many regions of the world, such as the less-developed countries. Therefore, identification of high-risk population is particularly important. Our study observed that plasma levels of SNHG1 at 12 months before diagnosis was sufficient to distinguish postmenopausal osteoporosis patients from healthy controls. Therefore, plasma SNHG1 may serve as a predictive biomarker for postmenopausal osteoporosis. In addition, postmenopausal females with continuously reduced plasma level of SNHG1 may take proper therapies to avoid the development of postmenopausal osteoporosis. In addition, our study also showed that detection of plasma SNHG1 may guide the treatment of postmenopausal osteoporosis. Therefore, future studies may focus on the clinical values of plasma SNHG1 in the determination of different treatment strategies and the early diagnosis of high risk populations.

It has been well-established in cancer biology that SNHG1 can regulate cancer cell behaviors by sponging multiple miRNAs, such as miR-145, miR-195, miR-338, and miR-497 [21–25], which are critical players in the differentiation of osteoblast and osteoclast and dysregulation of these miRNAs is correlated with bone loss and osteoporosis [26]. Therefore, SNHG1 may interact with these miRNAs to participate in osteoporosis. Future studies are needed to test our hypothesis.

In conclusion, plasma levels of SNHG1 were reduced with menopause and the development of osteoporosis. Detection of SNHG1 may provide guidance for the diagnosis and treatment of postmenopausal osteoporosis.

## Abbreviations

AUC: area under the curve
BMD: bone mineral density
HPOST: healthy postmenopausal females
HPRE: healthy premenopausal females
lncRNA: long (>200 nt) non-coding RNA
POSTO: postmenopausal osteoporosis patients
ROC: receiveroperating characteristic
RT-qPCR: Quantitative real time polymerase chain reaction
SNHG1: small nucleolar RNA host gene 1
